# Retrospective Cohort Study of COVID-19 in Patients of the Brazilian Public Health System with SARS-CoV-2 Omicron Variant Infection

**DOI:** 10.3390/vaccines10091504

**Published:** 2022-09-09

**Authors:** Thiago B. Murari, Larissa Moraes dos Santos Fonseca, Hernane B. de B. Pereira, Aloísio S. Nascimento Filho, Hugo Saba, Fulvio A. Scorza, Antônio-Carlos G. de Almeida, Ethel L. N. Maciel, José F. F. Mendes, Tarcísio M. Rocha Filho, John R. David, Roberto Badaró, Bruna Aparecida Souza Machado, Marcelo A. Moret

**Affiliations:** 1 Modelagem Computacional, University Center SENAI/CIMATEC, Salvador 41650-010, BA, Brazil; 2 SENAI Institute of Innovation (ISI) in Health Advanced Systems, University Center SENAI/CIMATEC, Salvador 41650-010, BA, Brazil; 3 Departamento de Ciências Exatas e da Terra, Universidade do Estado da Bahia, Salvador 41150-000, BA, Brazil; 4 Disciplina de Neurociência, Escola Paulista de Medicina, Universidade Federal de São Paulo (EPM/UNIFESP), São Paulo 04021-001, SP, Brazil; 5 Departamento de Engenharia de Biossistemas, Universidade Federal de São João del-Rei (UFSJ), São João del-Rei 36307-352, MG, Brazil; 6 Laboratório de Epidemiologia, Universidade Federal do Espírito Santo, Vitória 29075-910, ES, Brazil; 7 Departamento de Física and I3N, Universidade de Aveiro, 3880 Aveiro, Portugal; 8 International Center for Condensed Matter Physics and Instituto de Física, Universidade de Brasília, Brasília 70910-900, DF, Brazil; 9 Faculty of Public Health, Harvard T.H. Chan School of Public Health, Boston, MA 02115, USA; 10 Department of Immunology and Infectious Diseases, Harvard Medical School, Boston, MA 02115, USA

**Keywords:** booster, vaccine effectiveness, COVID-19

## Abstract

Several vaccines against COVID-19 are now available, based on different techniques and made by different laboratories spread around the world. With the roll out of the vaccination process in an advanced stage in many countries, the reduced risk of hospitalization due to the Omicron variant relative to the Delta variant infection, despite the higher transmission risk of Omicron, may lead to a misinterpretation of the results, as infection by Omicron is associated with a significant reduction in severe outcomes and shorter hospitalization time than the Delta variant. We compared the in-hospital mortality due to the Omicron (Jan–Mar 2022) with Gamma (Jan 2021) and Delta (Oct–Dec 2021) variants of patients in the Brazilian public health system. This study also discusses the decrease in booster vaccine effectiveness in patients hospitalized due to the Omicron variant compared with the Delta variant. Without a remodeling of vaccines for new variants, booster doses may be necessary with a shorter time interval.

## 1. Introduction

With the emergence of the coronavirus disease 2019 (COVID-19) pandemic, caused by severe acute respiratory syndrome coronavirus 2 (SARS-CoV-2), the world witnessed an unprecedented and rapid effort for the rational design, development, and emergency approval of vaccines against the disease. By August 2022, more than 500 million cases of the disease worldwide are reported, of which approximately 6.5 million are deaths [1]. In Brazil, approximately 30 million cases and 600,000 deaths are confirmed [1]. However, according to the World Health Organization (WHO), the total number of deaths is likely to be higher than recorded, since the disease may be underreported in some regions and an unusually high number of deaths were recorded since the pandemic began [2]. The COVID 19 pandemic caused several impacts not only in the health field, but also devastating social and economic impacts. In addition to overloading the health systems and impacting even the provision of essential health services, the pandemic brought significant social and economic effects, as more than 100 million people are at risk of falling into extreme poverty [3,4]. For this reason, there is an unprecedented quest to develop safe and effective vaccines for preventing COVID-19 and mitigating the pandemic.

Among the strategies adopted to mitigate the impacts of the COVID 19 pandemic, such as social distance and lockdown policies, the strategy with the highest effectiveness and lowest economic impact is mass immunization [5,6]. Through vaccination it is possible not only to prevent infection, but also to prevent severe cases of the disease and death. It is esteemed that vaccination measures were able to prevent over 14 million deaths from COVID-19 worldwide during the first year of the beginning of immunization [7]. While several already available vaccines are based on well-known methodologies, such as the inactivated virus vaccine CoronaVaC (Sinovac Biotech) [8], others use relatively new approaches: non-human adenovirus AZD1222 (AstraZeneca) [9], recombinant human adenovirus Ad26.COV2.S (Janssen) [10], mRNA BNT162b2 (Pfizer and BioNTech) [11], and mRNA-1273 (Moderna) vaccines [12].

Due to their high versatility, mRNA-based vaccines were the first vaccine to be developed and receive emergency validation from WHO, a year after the pandemic began [13]. The rapid development and availability of the new vaccine technologies was only possible because of the availability of the SARS-CoV-2 gene sequence, as well as the elucidation of information related to its mechanism of cellular action [14]. The SARS-CoV-2 genome presents six functional open reading frames (ORFs), encoding a replicase (ORF1a/ORF1b), spike (S), and structural proteins envelope (E), membrane (M), and nucleocapsid (N). Moreover, it presents seven putative ORFs encoding accessory proteins. The main target of the vaccines available, and for which efficacy data are available, is the S-protein, since it plays an essential role in the entry of the virus into cells, and is the main target of antibodies capable of neutralizing the virus [15].

Despite the more than 10 billion vaccine doses administered around the world by 10 August 2022 [16], and the proven disease control measures, including mask wearing and adaptation in businesses and schools, new SARS-CoV-2 variants of concern (VOC) are still emerging. SARS-CoV-2, together with SARS-CoV and Middle East respiratory syndrome coronavirus (MERS-CoV), is a single-stranded and positive-sense RNA virus, and, for this reason, presents an intrinsic nature of evolution and gradual change, leading to genetic mutations and the consequent emergence of new variants [17]. Mutations in the spike protein gene changed the binding efficiency of the protein to host cells, as well as the immunogenicity of the virus, resulting in more invasive variants [18,19]. The latest examples of new SARS-CoV-2 variants are the Delta variant (B.1.617.2), designated VOC on May 11, 2021, and the Omicron variant (B.1.1.529), designated VOC on 26 November 2021 [20].

Delta variant was classified as VOC due to its increased transmissibility, with an increased viral load in an infected individual one thousand times higher relative to the original SARS-CoV-2 virus [21]. These attributes are associated with 30 mutations in the viral genome, when compared to the reference sequence of Wuhan-01, including S protein mutation P681R, in the furin cleavage site, mutation L452R in the receptor-binding domain (RBD), and the nucleocapsid mutation R203M. Regarding the Omicron variant, it is also spreading faster than any previous variant, surpassing Delta in early 2022. Its transmission risk is twice as high as that of Delta in non-household settings, and is particularly observed in transmission between unvaccinated individuals. This may indicate that briefer contact events may be sufficient for transmission. The Omicron variant also has an increased capacity to evade vaccine-triggered immunity [22]. To date, its genome possesses a compelling number of mutations among all SARS-CoV-2 variants, with 37 changes within the S protein, 15 of which are on the RBD, and within the N-terminal domain site [23,24]. Despite the higher transmissibility of Omicron [25], mitigation policies, such as social distancing and mask mandates, are shown to effectively reduce the impacts of outbreaks, as clearly shown by the measures adopted in European countries, and in US and Brazilian states [26], and can be implemented easily and rapidly to reduce the impact of the Omicron variant.

Infections by the Omicron variant result in a smaller hospitalization risk than the Delta variant [27,28], despite the higher transmission risk. This may lead to a misinterpretation of results, as infection by Omicron is associated with less severe outcomes and shorter hospitalization than the Delta variant. The present study compares in-hospital mortality by the Omicron variant during January through March 2022 with Gamma during Jan 2021 and Delta during October through December 2021, for patients in the Brazilian public health system.

We discuss the decrease in the booster vaccine effectiveness (VE) of hospitalized patients for infections of Omicron, compared with the Delta variant. Feikin et al. published a systematic review of the VE considering the period from 17 June 2021 to 2 December 2021, but without data for the Omicron variant [29]. We stress the fact that a rational design of vaccination considering all variants and the respective VE is important for an effective mitigation of the current pandemic [30], with the side effect of lowering the probability of the emergence of yet another new variant, which tend to emerge in countries with low vaccination coverage.

## 2. Materials and Methods

This is a retrospective cohort study of COVID-19 hospitalization in the Brazilian public health system of 959,812 SARS-CoV-2-infected patients from a multicenter, nationwide database in Brazil from 1 January 2021 to 23 March 2022. The limitations of this study are: I. the Brazilian public health system represents 70% of all hospital beds in the country, and II. the rough dataset presented some data gaps, so we considered only data where the outcome of death or survival is given.

We tested whether the Omicron cohort reduced severity in outcomes that differed from the Gamma and Delta cohorts within hospitalized patients in the Brazilian public health system. The vaccination status documented in hospitalized patients (CoronaVaC, AZD1222, BNT162b2, and Ad26.COV2.S) and age were used in the evaluation (Table S1). Separate analyses were performed on patients stratified into three age groups (0–17 years old), adults (18–60 years old), and elderly individuals (over 60 years old). The in-hospital mortality outcome was examined in the studied time window. COVID-19 hospitalizations in the period was tested for normality (Figures S1 and S2)

## 3. Results

**Figure 1:**
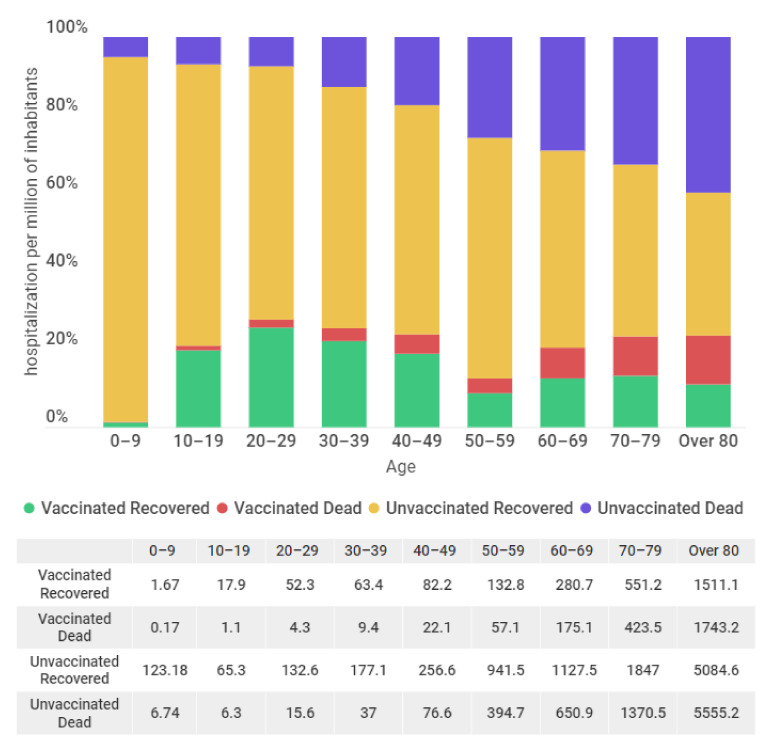
Hospitalizations and in-hospital deaths per million inhabitants between 1 January 2022 and 23 March 2022, according to vaccination status and age group. Unvaccinated included partially vaccinated individuals.

Figure 1 shows the number of hospitalized individuals per million inhabitants for the period from 1 January to 23 March 2022, according to vaccination status. This finding clearly shows the vaccine effectiveness, even without considering immunosenescence. In summary, unvaccinated individuals are approximately twice the number of vaccinated individuals among hospitalized individuals per million. For instance, if we observe the 60–69 age group, 280.7 hospitalized individuals per million inhabitants were fully vaccinated and recovered from COVID-19, while 175.1 hospitalized individuals per million inhabitants were fully vaccinated but died. Between the unvaccinated individuals, 1127.5 hospitalizations per million inhabitants recovered and 650.9 hospitalizations per million inhabitants died.

Hospitalization is significantly higher in unvaccinated or only partially vaccinated individuals. The number of hospitalized individuals increases, and the number of recovered people significantly decreases, with aging. The number of people who died is practically the same as those who recovered after hospitalization for the age group over 80. The vaccination for 5 to 10-year-olds only started in Brazil on 14 January 2022. Currently, the complete vaccination of this age group is approximately 85%.

Unvaccinated individuals were most likely to be hospitalized and die from COVID-19 from October to December 2021 (with dominance of the Delta variant) and January to March 2022 (with the Omicron variant dominant). The number of hospitalizations and deaths for the second period was 3.6 and 4.2 times higher when compared to the early period (Tables S2–S4).

Compared to late 2021, booster VE against severe outcomes for infections of the Omicron variant decreased 16.7% on average. CoronaVac VE decreased 15.32%, from 96.93% (95% confidence interval (CI: 96.35–97.51)) to 81.62% (95% CI: 80.99–82.23) when compared with late 2021. AZD1222 decreased 23.77%, from 96.56% (95% CI: 96.13–97.12) to 72.79% (95% CI: 72.20–73.37), and Ad26.COV2.S decreased 29.28% from 87.73% (95% CI: 83.65–91.80) to 58.44% (95% CI: 53.43–63.46). BNT162b2 VE decreased 7.52% with the Omicron variant, from 98.35% (95% CI: 97.52–99.18) to 90.84% (95% CI: 89.92–91.75), presenting the highest VE when compared to the other vaccines (Table 1).

**Figure 2:**
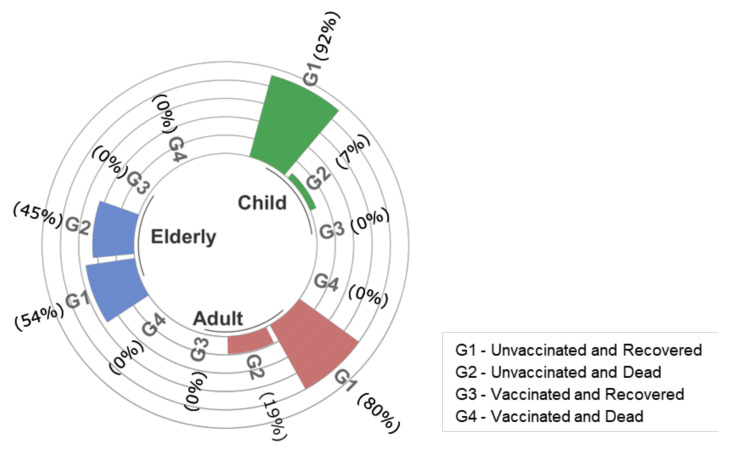
In-hospital mortality and recovery for January 2021 with the predominance of the Gamma variant infections among the hospitalized patients. “G1” stands for unvaccinated and recovered patients, “G2” for unvaccinated and dead, “G3” for vaccinated and recovered, and “G4” for vaccinated and dead. Age was stratified into three groups: child (0–17 years old), adult (18–59 years old), and elderly (over 60 years old).

**Figure 3:**
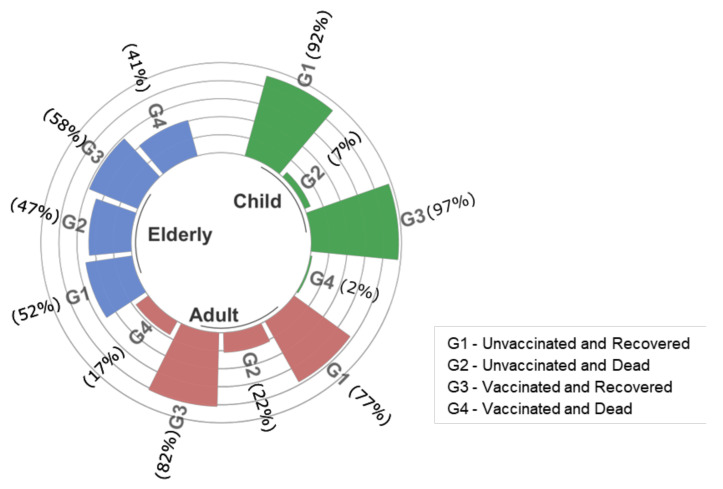
In-hospital mortality and recovery for October to December 2021 with the predominance of Delta. “G1” stands for unvaccinated and recovered patients, “G2” for unvaccinated and dead, “G3” for vaccinated and recovered, and “G4” for vaccinated and dead. Age was stratified into three groups: child (0–17 years old), adult (18–59 years old) and elderly (over 60 years old).

**Figure 4:**
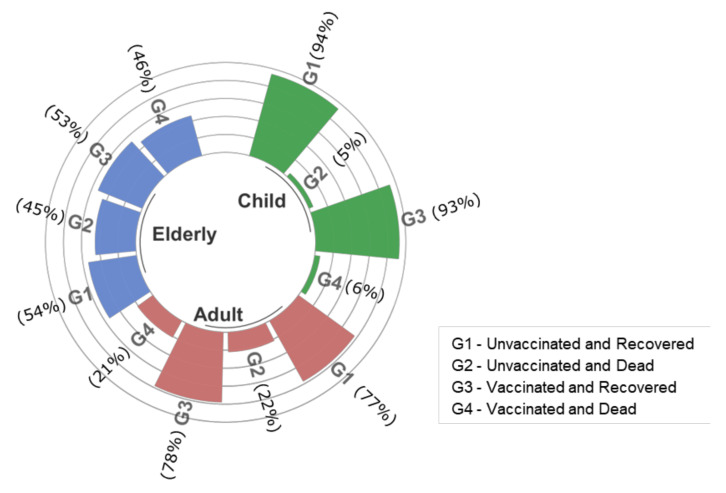
In-hospital mortality and recovery from January to March 2022 with the predominance of the Omicron variant. “G1” stands for unvaccinated and recovered patients, “G2” for unvaccinated and dead, “G3” for vaccinated and recovered, and “G4” for vaccinated and dead. Age was stratified into three groups: child (0–17 years old), adult (18–59 years old) and elderly (over 60 years old).

Figure 2, Figure 3 and Figure 4 show the profile of in-hospital mortality and recovery for January 2021 (Figure 2), with predominance of the Gamma variant, October to December 2021 (Figure 3) with the predominance of Delta, and January to March 2022 (Figure 4) with the predominance of the Omicron variant, for children and adolescents (0–17 years old), adults (18–59 years old), and the elderly (over 60 years old), in vaccinated and unvaccinated patients. Regardless of the variant, once hospitalized, the proportion of recovery and death outcomes was approximately the same in all age groups. Moreover, for all variants considered here, the proportion of recovered children was higher than that of adults. The attack rate obtained from epidemiological models [31], serological surveys [32,33], or estimates obtained from average infection fatality ratio must be considered with care due to the higher reinfection rate by the Omicron variant.

Regarding child hospitalization, the results of an evaluation of this population divided into two subgroups (0–9 years old and 10–17 years old) demonstrate that there are approximately four times more hospitalizations of children between 0 and 9 years old than in the age group between 10 and 17 years old for the Omicron variant (Table S5). For the subgroup between 0 and 9 years old, the mortality rates for unvaccinated and vaccinated children were 5.12% (95% CI: 4.76–5.47) and 0.13% (95% CI: 0.07–0.19), respectively. For the subgroup between 10 and 17 years old, the mortality rates for unvaccinated and vaccinated children were 6.59% (95% CI: 5.89–7.29) and 1.51% (95% CI: 1.16–1.85), respectively. Finally, the mortality rate is much higher for unvaccinated children than for vaccinated children.

## 4. Discussion

Gamma and Delta variants were dominant during 2021, resulting in two overlapping outbreaks, with similar hospitalization rates for infections caused by each variant. By the end of 2021 the Omicron variant was detected and became the leading cause of hospitalization by January 2022. At the beginning of the pandemic, hospitalizations were, in a great proportion, elderly individuals (over 60 years old). In Brazil, approximately half of COVID-19 severe cases occurred in this age group, accounting for 73% of deaths up to September 2020 [34,35]. In March 2022, there was a proportion of 85% fully vaccinated individuals among the elderly population, while the proportion of vaccinated individuals less than thirty years old was much lower. Another relevant factor impacting the age profile among the hospitalized population is the reduced VE for the Omicron variant. Moreover, due to the initial limited availability of vaccines for COVID-19 in Brazil, only targeted people who belonged to risk groups, such as health workers, elderly individuals, indigenous populations, and institutionalized individuals were vaccinated. Vaccination by age started with those above 85 years and older, and was gradually reduced for younger groups, with the younger population remaining exposed to the virus for a longer period, with booster doses for these individuals administered only starting in September 2021.

Despite the proven efficacy of vaccines, and with a significant proportion of the population immunized, COVID-19 remains a public health concern, as the number of new hospitalizations and deaths due to the disease remains important. Between late 2021 and early 2022, the moving average of daily cases increased worldwide, with a peak of more than four million new cases in a single day on 26 January 2022 [16]. In Brazil, this number was close to 300,000 cases per day on 5 February 2022, with a strong impact on the healthcare system [16]. An important contributing factor to this situation was the emergence of the Omicron variant, first reported on 26 November 2021 in South Africa, and classified as a variant of concern (VOC) by the WHO later that month [36]. The Omicron variant was responsible for the majority of cases by January 2022 and was estimated to be 3.2 times more transmissible than the Delta variant [37].

To date, 12 COVID-19 vaccines are granted for emergency use by the WHO, preventing SARS-CoV-2 infection, and thus mitigating the health and socioeconomic impacts caused by the pandemic [38]. In Brazil, the National Health Surveillance Agency (Anvisa) approved four of these vaccines: the inactivated virus vaccine CoronaVaC (Sinovac Biotech), the mRNA vaccine BNT162b2 (Pfizer and BioNTech), the non-human adenovirus vaccine AZD1222 (AstraZeneca), and the recombinant human adenovirus Ad26.COV2.S (Janssen) vaccine. Most of the approved and available vaccines for COVID-19 target the Spike (S) glycoprotein site from the original SARS-CoV-2 (Wuhan-01 strain) for neutralizing antibodies, due to its role in the virus infection and in the adaptive immune response [39].

Studies demonstrated that the Omicron variant mutation in its S glycoprotein epitope, including mutations within the receptor-binding domain (RBD), allows it to evade the antibody response and thus threatens measures to contain the infection. Such mutations result in resistance to neutralizing antibodies and are associated with reduced vaccine effectiveness. However, it is not completely clear to what extent T-cells are able to recognize this variant. The existing vaccines, even those capable of reducing the risk of hospitalization and death, may not be able to provide proper protection against Omicron variant infection, as its protection drops to less than 40% a few months after immunization [40,41]. Booster doses are proven to be an effective alternative in reducing new COVID-19 cases. It is important to highlight that information on the duration of vaccine-induced antibody responses is fundamental for deciding vaccine doses, including booster doses, contributing to the planning of infection prevention and control measures [42].

Evidence points to the critical relevance of booster doses in reducing hospitalizations. The first booster doses in Brazil were administered 180 days after the second dose (or a single dose for the Janssen vaccine), but this delay was reduced to 120 days. Based on the available data, the optimal delay is between 105 and 120 days (Table S2 and Table S3).

In vivo studies conducted with the CoronaVac and BNT162b2 vaccines with different vaccine regimes show that Omicron neutralization is weak or undetectable after complete immunization with two doses. Moreover, such studies and reports demonstrate that the booster dose can effectively prevent infection, hospitalizations, and deaths from COVID-19 [43,44,45]. As pointed out above, for both the CoronaVac and BNT162b2 vaccines, studies show that it is important to consider the interval between the second dose and the booster dose, as immunity declines over time [46].

Regarding other vaccine platform technologies, such as adenoviral vector vaccines, it was also shown that for the vaccine AZD1222, a third dose booster significantly increased the antibody levels against the Omicron variant [47]. Serum from individuals collected one month after receiving the booster dose was able to neutralize the action of the Omicron variant at levels similar to those observed one month after the second dose against the Delta variant, while two doses of AZD1222 yielded effective protection against the Delta variant [30]. It is important to mention that infection with the Omicron variant is associated with diseases that present less severe symptoms [27]. However, the reason for this is still unknown. This can be attributed to the fact that the various mutations in the viral genome potentially attenuated its virulence by affecting its replication and fusion efficiency with the host cell, which may partially explain these findings [48].

Although the Omicron variant is less lethal in the population, it did not differ much for the elderly population, who remain at risk due to the natural age-related decline in VE. In contrast, the VE behavior decreased for hospitalized children in the 0 to 17 years old group with the omicron variant (Table S2 and Table S3). A cross-sectional analysis of the hospitalizations and in-hospital deaths between 1st January 2022 and 23 March 2022 may suggest that the vaccination schedule should be updated to use only vaccines of a similar class of BNT162b2 to immunize elderly people.

Brazil has no mass testing policy, with self-tests approved only at the end of February 2022, but with no planned availability in the public health sector, where all treatments and medications are free. Additionally, there is no policy for the incorporation of effective drugs for the treatment of COVID-19 in the public health sector up to March 2022, which must be compared to the United Kingdom, where these drugs were responsible for the change in hospitalization outcomes in groups with a greater probability of evolving severity, such as elderly individuals, when treated in the first few days of symptoms [49]. Due to the protection guaranteed by the vaccines and the characteristics of the Omicron variant, which causes milder symptoms, the number of deaths has not grown at the same rate as the number of cases.

## 5. Conclusions

The Omicron variant is less lethal than Delta in the population, but it did not differ much for the elderly population who remain at risk due to the natural age-related decline in vaccine effectiveness. In contrast, the vaccine effectiveness decreased for hospitalized children in the 0 to 17 years old group with the omicron variant. In fact, the scenario is worst for children aged 6 months to 3 years, because COVID-19 killed more than three times as many people as the sum of all deaths in this age group over the last decade from diseases that were preventable by vaccines in 2020 and 2021, such as neonatal tetanus, diphtheria, polio, and others [50]. This age group of children between 6 months and 3 years old represents approximately two in five children under 5 years old who died with COVID-19 in the first two years of the pandemic [50], and we have no perspective on the vaccination for this age group in Brazil, a condition currently approved by the US Centers for Disease Control and Prevention.

Evidence also points to the critical relevance of booster doses in reducing hospitalizations. The first booster doses in Brazil were administered 180 days after the second dose (or a single dose for the Janssen vaccine), but this delay was reduced to 120 days. Based on the available data, the optimal delay is between 105 and 120 days.

Considering that the aged population in Brazil received the CoronaVac or AZD1222 vaccines as a primary vaccination regimen, the present discussion strongly reinforces the need for the increased protection of this population up to the end of 2022, preferably integrated with the influenza vaccination campaign that is already consolidated by the public immunization plan in Brazil. Finally, the analysis shows that, regardless of age group, the death ratio among hospitalized people remains the same for any of the studied periods.

## Figures and Tables

**Table 1:** Booster VE against SARS-CoV-2 for two different periods of time, without considering the immunosenescence. Incidence is shown per million of inhabitants.

Vaccine	Period	Hospitalizations	Hospitalizations	VE (95% CI)
		Fully Vaccinated	Booster	Booster
CoronaVac	Oct–Dec 21	19.29	0.59	96.93% (96.35–97.51)
	Jan–Mar 22	87.64	16.11	81.62% (80.99–82.23)
AZD1222	Oct–Dec 21	38.92	1.33	96.56% (96.13–97.12)
	Jan–Mar 22	128.03	34.83	72.79% (72.20–73.37)
BNT162b2	Oct–Dec 21	5.35	0.08	98.35% (97.52–99.18)
	Jan–Mar 22	24.18	2.21	90.84% (89.92–91.75)
Ad26.COV2.S	Oct–Dec 21	1.33	0.16	87.73% (83.65–91.80)
	Jan–Mar 22	2.16	0.90	58.44% (53.43–63.46)
Missing manufacturer information	Oct–Dec 21	0.89	0.15	83.19% (77.03–89.34)
	Jan–Mar 22	6.87	1.67	75.57% (72.90–78.24)

## Data Availability

In this study, we followed the Brazilian Personal Data Protection General Law. The anonymized data are publicly available at https://opendatasus.saude.gov.br/dataset/srag-2021-e-2022 downloaded on 24 March 2022. Any information for assessing the databases must be addressed to the Brazilian Ministry of Health at https://datasus.saude.gov.br/ and requests can be addressed to dadosabertos@saude.gov.br. Accessed on 24 March 2022.

## Supplementary Materials

The following supporting information can be downloaded at: https://www.mdpi.com/article/10.3390/vaccines10091504/s1, Table S1: Vaccination percentage of the Brazilian population stratified into three age groups; Table S2: Number of hospitalizations and deaths from Oct to Dec 2021 stratified into three age groups; Table S3: Number of hospitalizations and deaths from Jan to Mar 2022 stratified into three age groups; Table S4: Death of unvaccinated people (only hospitalized people analyzed); Table S5: Death of vaccinated and unvaccinated children (only hospitalized people analyzed); Figure S1: Normality test for COVID-19 hospitalizations from October 2021 to December 2021; Figure S2: Normality test for COVID-19 hospitalizations from January 2022 to March 2022.
