# Supplementary material for: Retrospective Cohort Study of COVID-19 in Patients of the Brazilian Public Health System with SARS-CoV-2 Omicron Variant Infection
Source: Vaccines (Basel). 2022 Sep 9;10(9):1504. doi: 10.3390/vaccines10091504 (PMC9500832; doi:10.3390/vaccines10091504)
Supplement: Supplementary file 1 [file vaccines-10-01504-s001.zip › vaccines-1878177-supplementary.pdf]

Table S1. Vaccination percentage of the Brazilian population stratified into three age groups.

Age	Vaccination schedule					
	Oct–Dec 2021 (%)			Jan–Mar 2022 (%)		
	Partial	Fully	Booster	partial	fully	booster
0–17	31.5	20.8	0.2	48.4	27.2	1.5
18–59	87.7	77.8	8.7	89.2	80.7	33.0
60+	88.2	87.3	52.4	88.4	87.6	64.1

Table S2. Number of hospitalizations and deaths from Oct to Dec 2021 stratified into three age groups.

Vaccine	Age	Hospitalized					Dead				
		Partially Vaccinated	Fully Vaccinated			Booster	Partially Vaccinated	Fully Vaccinated			Booster
			>180 days	<180 days	<120 days			>180 days	<180 days	<120 days	
CoronaVac	0–17	3	1	9	8	0	1	0	0	0	0
	18–59	215	335	617	387	7	39	39	87	53	0
	Over 60	202	357	2075	861	25	91	156	789	289	13
AZD1222	0–17		1	9	8	0	1	0	0	0	0
	18–59	378	55	1498	1201	7	67	1	318	254	0
	Over 60	285	3407	1926	105	110	132	1544	747	46	60
BNT162b2	0–17	46	3	14	13	0	3	0	1	1	0
	18–59	330	56	361	319	1	64	6	63	57	1
	Over 60	63	371	87	51	4	31	134	36	23	2
Ad26.COV2.S	0–17	0	0	1	1	0	0	0	0	0	0
	18–59	208	11	4	4	0	51	0	1	1	0
	Over 60	15	4	6	5	0	5	2	3	3	0
Missing manufacturer information	0–17	7	0	0	0	0	3	0	0	0	0
	18–59	23	5	42	22	2	4	0	6	0	1
	Over 60	13	51	37	13	5	7	17	11	5	1

Table S3. Number of hospitalizations and deaths from Jan to Mar 2022 stratified into three age groups.

Vaccine	Age	Hospitalized					Dead				
		Partially Vaccinated	Fully Vaccinated			Booster	Partially Vaccinated	Fully Vaccinated			Booster
			>180 days	<180 days	<120 days			>180 days	<180 days	<120 days	
CoronaVac	0–17	3	7	13	9	6	0	1	0	0	0
	18–59	563	696	4536	1153	304	174	184	1115	230	81
	Over 60	639	5632	2219	313	1387	344	2659	846	140	645
AZD1222	0–17	28	15	5	3	2	2	0	0	0	0
	18 - 59	350	760	1376	526	159	81	150	210	85	26
	Over 60	765	14693	633	376	4447	391	7172	304	172	2147
BNT162b2	0–17	120	17	135	128	2	20	4	8	7	0
	18–59	526	144	1771	1042	95	112	19	289	136	27
	Over 60	180	1271	336	189	36	82	488	124	78	15
Ad26.COV2.S	0–17	0	4	5	2	0	0	1	0	0	0
	18–59	209	10	45	38	12	33	3	7	5	2
	Over 60	36	19	29	9	2	15	9	13	4	1
Missing manufacturer information	0–17	7	1	8	7	4	0	0	1	1	0
	18–59	57	59	195	80	12	8	10	34	12	2
	Over 60	76	566	116	49	35	37	257	47	18	18

Table S4. Death of unvaccinated people (only hospitalized people analyzed).

Age	Jan 2021		Oct–Dec 2021		Jan–Mar 2022	
	Death (%)	95% CI	Death (%)	95% CI	Death (%)	95% CI
0-17	7.70	6.25–9.16	7.29	4.62–7.36	5.74	5.56–9.83
18-59	19.43	19.03–19.84	22.34	19.16–21.10	22.09	18.73–22.87
60+	45.08	44.63–45.52	47.65	43.23–45.71	45.23	45.08–49.31

Table S5. Death of vaccinated and unvaccinated children (only hospitalized people analyzed).

	Unvaccinated				Vaccinated		
	Age	Recovered	Dead	Dead (95% CI)	Recovered	Dead	Dead (95% CI)
**Jan 2021**	0–9	642	47	6.82% (5.86%–7.78%)	-	-	-
	10–19	255	34	11.76% (9.87%–13.66%)	-	-	-
**Out–Dez 2021**	0–9	620	51	7.42% (6.42%–8.42%)	-	-	-
	10–19	287	27	7.54% (6.15%–8.94%)	-	-	-
**Jan–Mar 2022**	0–9	3618	198	5.12% (4.76%–5.47%)	49	5	0.13% (0.07%–0.19%)
	10–19	859	83	6.59% (5.89%–7.29%)	299	19	1.51% (1.16%–1.85%)

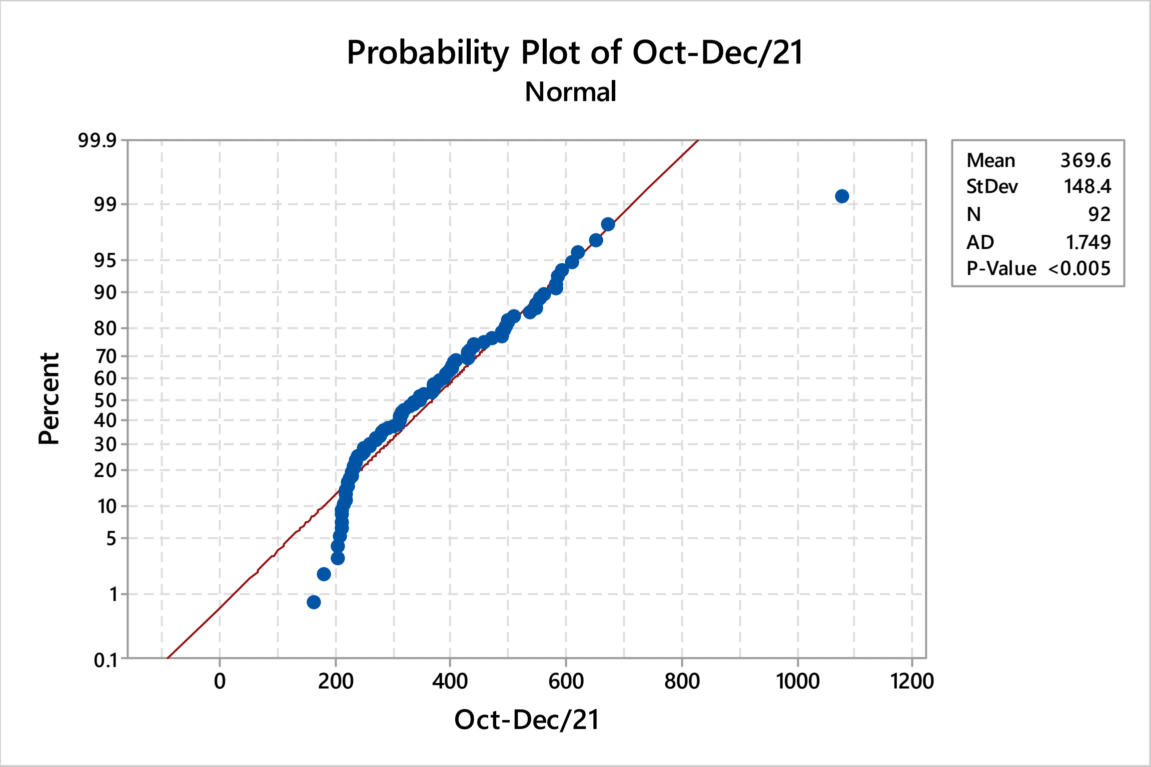

Figure S1. Normality test for COVID-19 hospitalizations from October 2021 to December 2021.

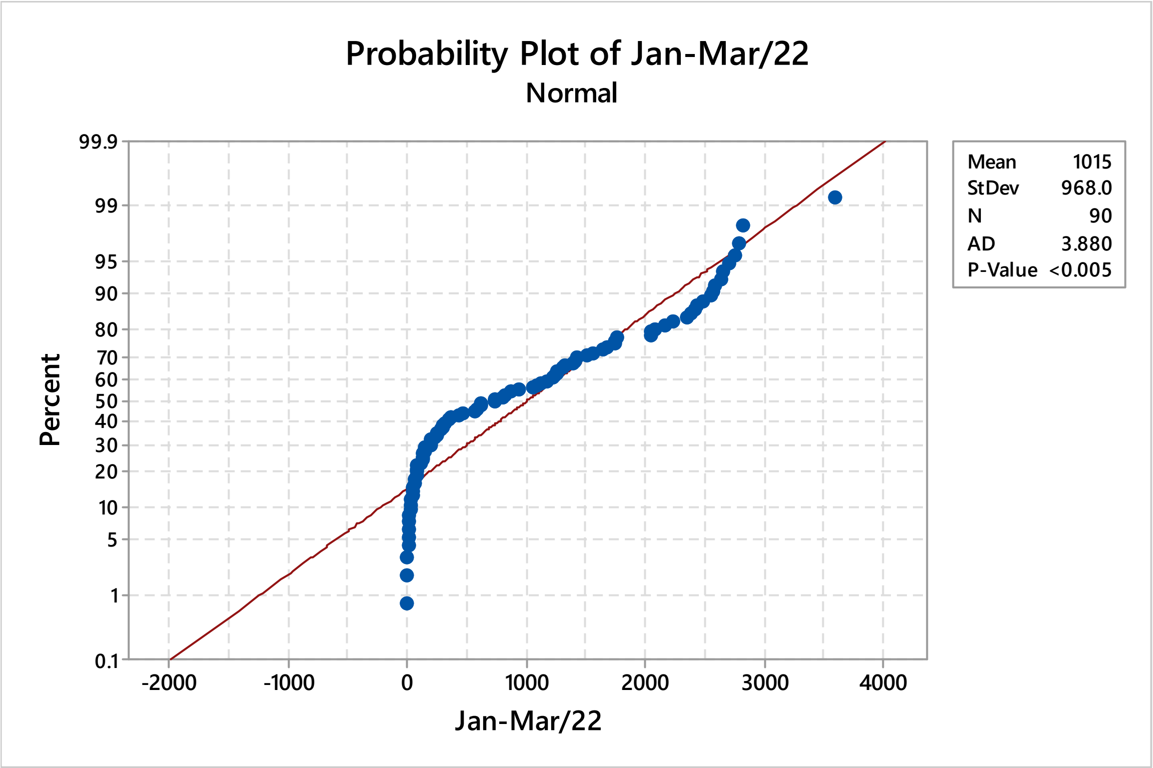

Figure S2. Normality test for COVID-19 hospitalizations from January 2022 to March 2022.
